# Annealing and N_2_ Plasma Treatment to Minimize Corrosion of SiC-Coated Glass-Ceramics

**DOI:** 10.3390/ma13102375

**Published:** 2020-05-21

**Authors:** Chaker Fares, Randy Elhassani, Jessica Partain, Shu-Min Hsu, Valentin Craciun, Fan Ren, Josephine F. Esquivel-Upshaw

**Affiliations:** 1 Chemical Engineering Department, University of Florida College of Engineering, Gainesville, FL 32611, USA; c.fares@ufl.edu (C.F.); randyelhassani@ufl.edu (R.E.); jpartain3@ufl.edu (J.P.); fren@che.ufl.edu (F.R.); 2 Department of Restorative Dental Sciences, University of Florida College of Dentistry, Gainesville, FL 32610, USA; shuminhsu@ufl.edu; 3 Plasma and Radiation Physics, National Institute for Laser, Laser Department, RO-077125 Bucharest, Romania; valentin.craciun@inflpr.ro

**Keywords:** plasma treatment, coating, corrosion, pore sealing, glass ceramic, biomaterials

## Abstract

To improve the chemical durability of SiC-based coatings on glass-ceramics, the effects of annealing and N_2_ plasma treatment were investigated. Fluorapatite glass-ceramic disks were coated with SiC via plasma-enhanced chemical vapor deposition (PECVD), treated with N_2_ plasma followed by an annealing step, characterized, and then immersed in a pH 10 buffer solution for 30 days to study coating delamination. Post-deposition annealing was found to densify the deposited SiC and lessen SiC delamination during the pH 10 immersion. When the SiC was treated with a N_2_ plasma for 10 min, the bulk properties of the SiC coating were not affected but surface pores were sealed, slightly improving the SiC’s chemical durability. By combining N_2_ plasma-treatment with a post-deposition annealing step, film delamination was reduced from 94% to 2.9% after immersion in a pH 10 solution for 30 days. X-ray Photoelectron spectroscopy (XPS) detected a higher concentration of oxygen on the surface of the plasma treated films, indicating a thin SiO_2_ layer was formed and could have assisted in pore sealing. In conclusion, post-deposition annealing and N_2_ plasma treatment where shown to significantly improve the chemical durability of PECVD deposited SiC films used as a coating for glass-ceramics.

## 1. Introduction

Within the dental industry, glass-ceramics are often used in fixed dental prostheses such as crowns, veneers, and bridges to restore missing parts of a patient’s dentition. Over time, these glass-ceramics have been shown to undergo corrosion due to the caustic environment of the oral cavity [1,2,3,4,5,6,7,8]. Previous clinical studies have not only confirmed this, but demonstrate that the severity of the corrosion process is strongly correlated to the pH levels that the ceramics are exposed to [9,10]. Consequently, these corrosion mechanisms lead to a reduced fracture strength of the glass ceramic and cause a roughened surface topography [11,12,13,14]. When a ceramic restoration roughens, the increased wear on adjacent oral structures can cause additional damage and also lead to additional plaque accumulation [15,16].

To improve the chemical durability of these glass-ceramics, intrinsic and extrinsic modifications have been studied. For intrinsic modifications, a number of reports have focused on improving the glass-ceramic chemical stability by altering the ceramic’s composition, incorporating additional oxides such as Al_2_O_3_, CaO, or K_2_O, or adding calcium phosphates or fluorine to the structure [17,18,19]. Unlike intrinsic modifications, external modifications do not alter the composition of the glass-ceramic but rather function by applying an additional layer to the surface to protect the ceramic material [20,21,22]. For example, Esquivel-Upshaw et al. demonstrated that glazed ceramics are more corrosion-resistant than their un-glazed counterparts [1].

Out of many potential materials that could protect a glass-ceramic from corrosion, silicon carbide (SiC) is drawing significant interest due to this material’s high strength, lack of reactivity to the oral environment, and ease of deposition [23,24,25,26,27,28,29,30,31,32]. There have been several recent reports demonstrating the efficacy of SiC-based coatings on dental ceramics [31,33]. Chen et al. reported on the effects of deposition conditions on the coating properties and illustrated that the SiC could be modulated to match any tooth shade required for a fixed dental prostheses procedure [31]. Hsu et al. reported that coating glass-ceramics with SiC significantly reduced surface corrosion when placed in varying pH solutions used to mimic the oscillating caustic environment of a patient’s mouth [33]. In addition to SiC’s durability, SiC has also shown favorable biocompatibility in various applications [34,35,36,37,38,39,40]. Although these initial reports have demonstrated the promise of SiC-based ceramic coatings, significant work is still needed to optimize the chemical durability of the SiC film and ensure the coating does not delaminate over time.

One of the primary causes of SiC delamination is due to the porosity of the Silicon Carbide film [38,41,42]. Porosity has been purposely introduced into materials such as SiC within the microelectronics industry in order to achieve a future-generation of ultralow-*k* (ULK) materials having a dielectric constant near or less than 2.2 [42,43,44,45]. One of the downsides of porosity within SiC films is the ability for a liquid or impurity to diffuse through the pores and cause portions of the film to delaminate [45,46,47,48,49]. Although we are not interested in the electrical properties of SiC films, we can expound on knowledge from the microelectronics industry to increase the chemical robustness of SiC protective coatings.

To prevent SiC film delamination, several approaches have been studied including the deposition of a thin dielectric liner [42,50], an ion beam treatment [51], film densification through annealing [31], and the modification of the SiC surface using plasma treatment [52,53]. In this study, we report on the effects of annealing and plasma treatment on SiC films used as a protective coating for glass-ceramics. To accomplish this goal, we prepared SiC-coated ceramics using several different processing techniques, characterized the resulting SiC film for each technique, and then immersed the coated ceramic into a caustic solution to quantify any resulting delamination.

## 2. Materials and Methods

### 2.1. Specimens Preparation

Prior to SiC coating, fluorapatite glass-ceramic disks (Ivoclar Vivadent AG, Schaan, Liechtenstein, 12.6 X 2 ± 0.2 mm) were first polished using silicon carbide abrasive paper (Carbimet, Buehler, Lake Bluff, IL, USA). After polishing, the samples were cleaned by sonicating the disks in acetone in an ultrasonic bath, rinsing the disks with isopropyl alcohol, drying the disks with compressed nitrogen, and then finally, using ozone treatment to remove surface carbon contamination.

### 2.2. SiC Coating

After polishing and cleaning the ceramic disks, a plasma-enhanced chemical vapor deposition system (PECVD, PlasmaTherm 790, Saint Petersburg, FL, USA) was used to coat the disks with 20 nm of silicon dioxide (SiO_2_) and 200 nm silicon carbide (SiC) on both sides. The purpose of the SiO_2_ film was to improve adhesion between the ceramic and SiC [31]. The precursors for the SiO_2_ deposition were silane (SiH_4_) and nitrous oxide (N_2_O). After the SiO_2_ deposition, methane (CH_4_) and silane (SiH_4_) were the precursors used for silicon-carbide deposition. The deposition rates for SiO_2_ and SiC were 330 Å /min and 170 Å/min, respectively, at a temperature of 300 °C [31]. The variation in the deposition rate was determined to be within 1.3% of the total SiO_2_/SiC film thickness of 220 nm. The temperature within the PECVD remained within 5 °C of the set-point throughout the deposition process.

### 2.3. Post-Deposition Processing

After the glass-ceramic disks were coated with SiC, some were left as-deposited, some were annealed, some were plasma-treated, and some were plasma-treated followed by an annealing step. The plasma treatment processing steps were performed within the PECVD directly after SiC deposition on each side. For N_2_ plasma treatment, 200 sccm of N_2_ was used at an operating power of 400 W and temperature of 300 °C. The temperature during the plasma treatment remained within 5 °C of the set-point, and the nitrogen flow rate deviated by less than 0.2 sccm during the treatment process. For the post-deposition annealing steps, the SiC-coated disks were removed from the PECVD and placed into a tube-furnace. The coated disks were annealed in ambient nitrogen at 400 °C for 12 h.

### 2.4. Experimental Design

To determine the effects of annealing and N_2_ plasma treatment separately, four groups were utilized in this study. The four subgroups investigated in this study were fluorapatite glass-ceramic disks that have been coated with SiO_2_/SiC and; (i) have not been processed further (reference) (ii) annealed at 400 °C for 12 h, (iii) treated with an N_2_ plasma for 10 min, and (iv) annealed at 400 °C for 12 h and N_2_ plasma treated for 10 min. The annealing temperature and time were selected in order to reduce the intrinsic film stress of the SiC. In pilot studies, temperatures below 400 °C did not adequately reduce film stress, whereas temperatures significantly higher than 400 °C caused the SiC stress to become tensile, reducing the adhesive properties. The plasma treatment conditions were selected based on previous reports, pilot studies, and the limitations of the equipment utilized for this study [52]. After fabrication, each disk was characterized (additional information below) and then placed into a tube containing 15 mL of a pH 10 buffer solution (Potassium Carbonate–Potassium Borate–Potassium hydroxide buffer, SB116-500, Fisher Chemical). Next, the tubes containing the coated disks were set into a water bath that rotated at 50 oscillations per minute at a temperature of 80 °C (water bath, TSBS40, Techne USA). The temperature and timeframe of the corrosion study were chosen to simulate several years of degradation in a shorter amount of time. In a previous report, Hsu et al. justified the used of higher temperatures to simulate accelerated aging by deriving activation energies at various conditions [54]. For the stress-measurements and immersion results, three samples were utilized to take an average. For the Fourier-Transform Infrared Spectroscopy (FTIR) and photoelectron spectroscopy (XPS) data, analyses were performed across several points on the sample surface to ensure consistency.

### 2.5. Characterization Techniques

FTIR (Thermo Electron Magna 760, Waltham, MA, USA) was used to determine the chemical composition of the SiC after deposition, after annealing, and after plasma treatment.

A profilometer (Dektak 150, Veeco, St. Paul MN, Tucson, AZ, USA) was used to determine the film stress, σ_f_, of the SiC coating. The stress was calculated by measuring the curvature of a 2” silicon wafer with and without a SiO_2_/SiC coating for each of the studied conditions.

X-ray reflectivity (XRR) curves from the grown films were acquired in the 0.2–5 deg. range using an instrument (Empyrean from Panalytical, Eindhoven, Netherlands) working with a Cu anode (power settings of 45 kV and 40 mA) in a parallel beam geometry. The XRR curves were simulated using the Panalytical software packet Reflectivity™ and a model consisting of a three-layer structure: an interfacial layer between the deposited film and the substrate due to the Si native oxide, the deposited film, and a surface contamination layer accounting for surface oxidation when the films were exposed to ambient.

Scanning electron microscopy (SEM) was utilized to examine the surface morphology of uncoated glass-ceramic disks and SiC-coated glass ceramic disks were examined before and after a 28-day immersion in pH 10 buffer solution. The disks were sputter-coated with gold/palladium to mitigate charging effects and then analyzed using a field-emission SEM (FEI Nova 430, Hillsboro, OR, USA). The images were obtained at 5 kV.

A Physical Instruments ULVAC PHI XPS system (ULVAC PHI, Kanagawa, Japan) was used to determine the surface composition of the SiC for all conditions studied. The source power for this system was 300 W and a monochromatic Al X-ray source (1486 eV) was used. An electron pass energy of 93.5 eV was used for the survey scans along with an acceptance angle of 7°, take-off angle of 50°, and analysis spot diameter of 100 µm. The binding energy accuracy was within 0.03 eV, whereas the overall energy resolution of the XPS is approximately 0.1 eV. The C1s adventitious carbon peak at 284.8 eV was utilized for charge correction.

## 3. Results

**Figure 1:**
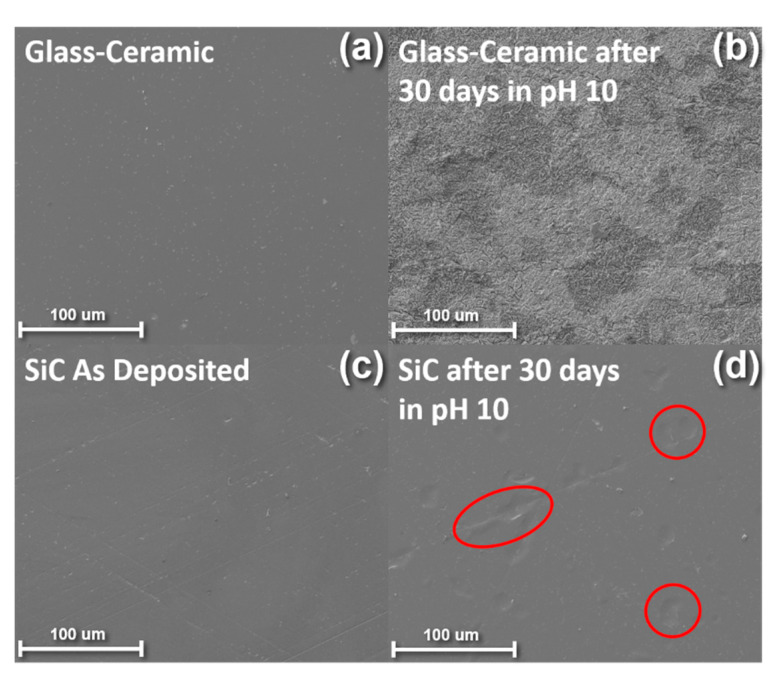
SEM images of (**a**) a fluorapatite glass-ceramic disk before corrosion (**b**) a fluorapatite glass-ceramic disk after corrosion (**c**) a SiC-coated fluorapatite glass-ceramic disk before corrosion and (**d**) a SiC-coated fluorapatite glass-ceramic disk after corrosion.

Figure 1 shows SEM images of an uncoated glass-ceramic disk before and after corrosion in a pH 10 buffer solution (a–b) and a SiC-coated ceramic disk before and after immersion in a pH 10 buffer solution (c–d). The uncoated fluorapatite glass-ceramic disks showed a significant increase in surface roughness after immersion. Ceramic corrosion can either occur through a total dissolution of the glass-ceramic network or through ionic exchange between network modifiers and ions in the surrounding solution [1]. In the case of the pH 10 buffer solution used in this study, total dissolution of the network-formers occurred, causing the increased surfaced roughness shown in Figure 1b. In contrast, Figure 1d illustrated that the SiC was successful in protecting the glass-ceramic from corrosion. These results agree with the previous literature reporting on the weight loss after corrosion of uncoated glass-ceramics as compared to SiC-coated glass-ceramics [33]. Andrews et al. examined the mechanism of corrosion for SiC in both acidic and basic conditions. In acidic conditions, a passivating layer of SiO_2_ is formed that prevents the dissolution of SiC, whereas in basic conditions, the SiC directly dissolves into SiO_3_^2−^ [55]. Another group compared the corrosive effects of SiC-coated glass-ceramics in pH 2, 7, and 10 buffer solutions and found that the pH 10 conditions exhibited the most severe corrosive effects [33]. Based on these previous findings, we have chosen a pH 10 buffer solution to evaluate our SiC film since this solution type demonstrated the harshest corrosive behavior. The regions circled in red in Figure 1d highlight areas of SiC that have delaminated from the glass-ceramic substrate after immersion in the pH 10 solution. Although, these results indicate that coating glass-ceramics in SiC could be an effective way to mitigate corrosion effects, further optimization is required to enhance the chemical durability of SiC films to prevent delamination and bubbling. To accomplish this task, the remainder of this report will focus on annealing and plasma treatment steps taken post-SiC deposition to improve SiC’s durability in caustic environments.

**Figure 2:**
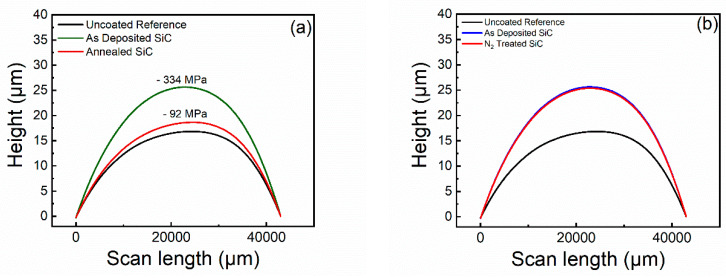
Curvature of a 2” silicon wafer coated before and after depositing 200 nm of SiC followed by (**a**) a post-deposition annealing at 400 °C for 12 h and (**b**) post-deposition plasma treatment with N_2_ for 10 min.

The film stress of PECVD-deposited SiC is well known to vary based on the deposition conditions, film thickness, and post-deposition processing steps [27,30,31,56]. This stress can either be tensile or compressive and greatly affect the adhesive properties of the SiC to the corresponding substrate. Therefore, the first metric we chose to evaluate in our study is the effect of post-deposition annealing and N_2_ plasma treatment on the film stress of the PECVD SiC protective coating. To accomplish this, the contours of several 2-inch silicon wafers were measured before and after the deposition of 200 nm of SiC followed by either annealing or plasma treatment. Figure 2 illustrates the effect of annealing and plasma treatment on the film stress of the SiO_2_/SiC coating. As shown in Figure 2a, the height of curvature across the wafer increased from 16 to 25 μm after SiC deposition. After annealing the same coated wafer, the height of curvature decreased from 25 to 18 μm. Figure 2b shows that the height of curvature for the N_2_ plasma-treated SiC remained constant after plasma treatment. The change in contour after each processing step can be related to film-stress by using the Stoney equation, shown below

(1) $$\sigma_{f}=\frac{E_{s}\times d_{s}^{2}}{6\left(1-\nu_{s}\right)}\times \frac{1}{d_{f}}\times \left(\frac{1}{R_{\mathrm{post}}}-\frac{1}{R_{\mathrm{pre}}}\right)$$

where ν_s_ and E_s_ Possion’s ratio and the Young’s modulus of the silicon wafer, respectively. R_pre_ and R_post_ are the radii of curvature before and after film deposition. Lastly, d_s_ is the thickness of the silicon wafer, and d_f_ is the thickness of the SiC film [57,58]. After fitting the data, the SiC film was determined to have a compressive stress of 334 MPa which was reduced to 92 MPa after annealing. This trend was also shown in a previous report by Chen et al. [49]. The specific mechanisms contributing to intrinsic stress within the SiC has been attributed to moisture absorption on pore walls, differences in thermal expansion coefficients, the reaction of H_2_O with Si−H, and strained Si−C bonds formed during PECVD deposition [59,60,61]. These strained bonds have a high reactivity with H_2_O and yield a compressive stress due to the repulsive forces created between opposing dipoles from H_2_O absorbed on the pore walls. Therefore, a minimization of these drivers could reduce the reactivity effects of the SiC when placed in an aqueous solution. The choice of temperature for the annealing process was chosen so that the resulting film stress remained compressive. Choice of temperature and time of the post-deposition step is, therefore, crucial in the final adhesive properties of the film. Unlike annealing, the N_2_ plasma-treatment step did not change the film stress of the deposited SiC film. This was expected as the N_2_ plasma treatment occurred directly after SiC deposition and at the same temperature as the SiC deposition. After understanding the effects of plasma treatment and annealing on the film stress, understanding any effects on the bulk chemistry and surface chemistry of the SiC from these processing steps is imperative.

**Figure 3:**
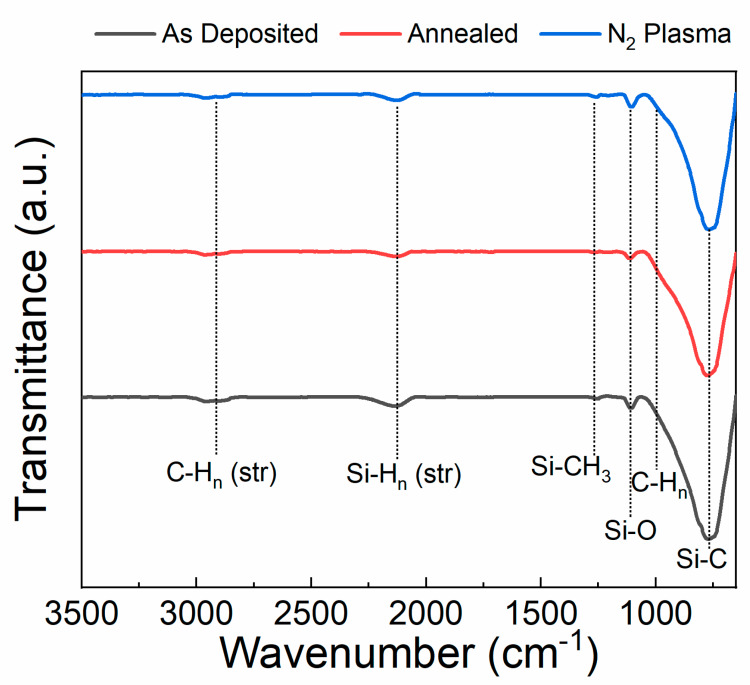
FTIR spectra of plasma-enhanced chemical vapor deposition (PECVD)-deposited SiC as-deposited, after 400 °C post-deposition annealing, and after 10 min of N_2_ plasma treatment.

Figure 3 shows FTIR spectra of the SiC films as-deposited, after annealing, and after plasma treatment. As seen in the FTIR spectra, the SiC films exhibits various peaks centered at ~790 cm^−1^, ~1010 cm^−1^, ~1110 cm^−1^, ~1250 cm^−1^, ~2070 cm^−1^, and ~2890 cm^−1^. The intense peak that appears ~790 cm^−1^ is attributed to Si−C stretching vibrations [62,63,64]. The shouldered peak located at 1010 cm^−1^ is due to C−H_n_ wagging. The peak observed ~1110 can be attributed to Si−O−Si stretching vibrations. At 1250 cm^−1^, a small peak corresponding to Si−CH_3_ bending can be observed [62]. The smaller peaks that appear near 2000–2200 cm^−1^ and 2890 cm^−1^ correspond to Si−H_n_ stretching mode vibrations (n = 1, 2), and stretching mode vibrations of C−H_2_ and C−H_3_, respectively [53,65,66]. For the annealed SiC sample, a small decrease in the relative intensity of the hydrogen-related peaks around 900 cm^−1^, 2000–2200 cm^−1^, and 2890 cm^−1^ can be observed. Additionally, a larger decrease is evident in Si−O−Si bonding, suggesting the annealing was sufficient to remove any absorbed moisture within the pores [49]. These results suggest an increase in the Si−C bond density after annealing, which is confirmed by comparing the relative peak intensities of the as-deposited and annealed films. The hydrogen incorporation in PECVD-deposited SiC contributes to a lower hardness compared to SiC grown using other methods [56]. For the N_2_ plasma-treated SiC, the bulk film composition remains similar to the as-deposited SiC. This result is in agreement with the film stress measurement in that the N_2_ plasma treatment primarily affects the surface of the SiC without altering the film’s bulk properties.

**Figure 4:**
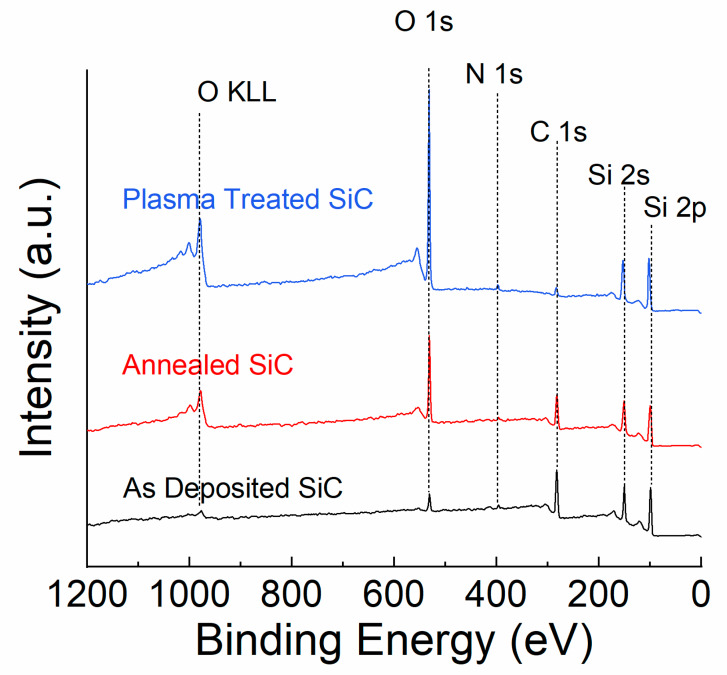
XPS survey scans of PECVD-deposited SiC as-deposited, after 400 °C post-deposition annealing, and after 10 min of N_2_ plasma treatment.

After understanding how the SiC’s bulk properties were affected by post-deposition annealing and N_2_ plasma treatment, XPS was utilized to understand the surface chemistry of the SiC after each processing condition. Figure 4 shows XPS survey scans of the SiC films as-deposited, after annealing, and after plasma treatment. After 10 min of N_2_ plasma treatment, the O 1s peak intensity increased significantly, whereas the C 1s peak intensity decreased significantly. The nitrogen concentration remained within the detection limits of the tool, indicating no nitrogen incorporation occurred during the plasma-treatment process. The higher concentration of oxygen on the surface of the SiC after N_2_ plasma treatment can give us an insight into how plasma treatment may be beneficial for chemical durability. As the energetic nitrogen ions bombard the SiC surface during plasma treatment, carbon is selectively etched from the surface, leaving a large concentration of silicon dangling bonds. Once the plasma-treated SiC samples are removed from under vacuum, the silicon dangling bonds quickly oxidize when exposed to the atmosphere, causing a thin SiO_2_ layer to form. In addition to the SiO_2_ cap layer formed after plasma treatment, several groups have reported the surface exposed to plasma treatment becomes densified due to the plasma breaking surface Si−H bonds [52]. After post deposition annealing, the O 1s peak increased to a smaller degree than the plasma-treated sample and the C 1s peak remained prominent. The atomic concentrations for all three processing conditions are shown in Table 1. Since FTIR data revealed a reduction of hydrogen-related bonds after annealing, silicon atoms previously attached to hydrogen near the surface were likely oxidized when exposed to the atmosphere. Lee et al. showed that when plasma-treating Hydrogen silsequioxane thin films, a surface-level SiO_2_ layer formed and a densified film was observed after plasma treatment [52]. Additionally, Lee showed that the thickness of the densified surface layer was a function of plasma power and plasma exposure time. With regards to SiC-based protective coatings, more work is needed to optimize the plasma-treatment conditions.

Table 2 shows the corresponding density of the SiC films as-deposited, after nitrogen plasma treatment for 10 min, after N_2_ plasma treatment followed by annealing, and after only annealing. The largest change in SiC density is due to annealing the film, which densified the film from 1.99 to 2.11 g/cm^3^. After N_2_ plasma treatment, the density of the SiC decreases 3.5% to 1.92 g/cm^3^. Similarly, the samples that were both N_2_ plasma treated and annealed showed a density that was approximately 2.4% lower than the samples that were exclusively annealed. The surface-roughness values measured by XRR were within 0.7 and 0.76 nm for all conditions studied, indicating that no major changes in roughness were detected after post-deposition annealing or plasma treatment. The measured XRR data is in agreement with the FTIR and XPS results. After annealing, intrinsic stress within the SiC film was reduced, densifying the film. For the plasma-treated samples, the slight decrease in density could be due to the surface-level SiO_2_ layer reducing the average density of the film and the selective removal of surface carbon after plasma exposure.

**Figure 5:**
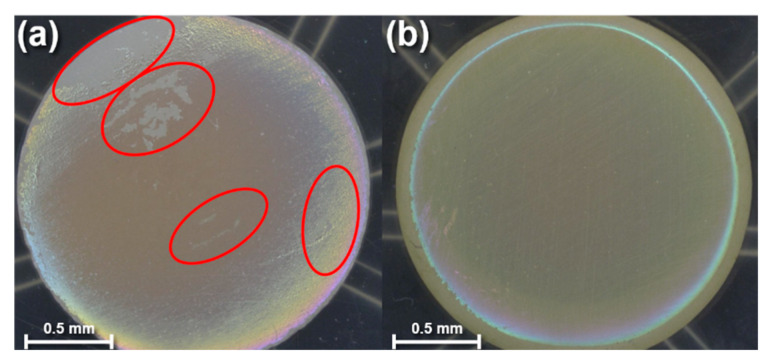
Visual microscope images of (**a**) an as-deposited SiC-coated glass-ceramic after being immersed into a pH 10 buffer solution at 80 °C for 30 days and (**b**) a N_2_ plasma-treated and post-deposition annealed SiC-coated glass-ceramic after being immersed into a pH 10 buffer solution at 80 °C for 30 days.

After characterizing the effects of N_2_ plasma treatment and post-deposition annealing on the PECVD-deposited SiC films, three disks per condition were immersed into a pH 10 buffer solution to quantify if the added processing steps reduced delamination and bubbling. Figure 5 shows optical microscope photos of fluorapatite glass-ceramic disks coated in SiC after immersion in a pH 10 buffer solution for 30 days. The disk in Figure 5a was coated in SiC and then directly immersed into the pH 10 buffer solution, whereas the disk in Figure 5b was plasma-treated with N_2_ for 10 min and then annealed before being placed into a pH 10 buffer solution. An optical surface-area analysis tool was utilized to quantify the amount of film peeling/bubbling after immersion. The as-deposited SiC-coated glass-ceramics exhibited an average of 94% of the film either peeling or bubbling after immersion. For the SiC-coated ceramic that was plasma-treated and annealed, the film delamination and bubbling was measured to be approximately 2.9% after immersion.

**Figure 6:**
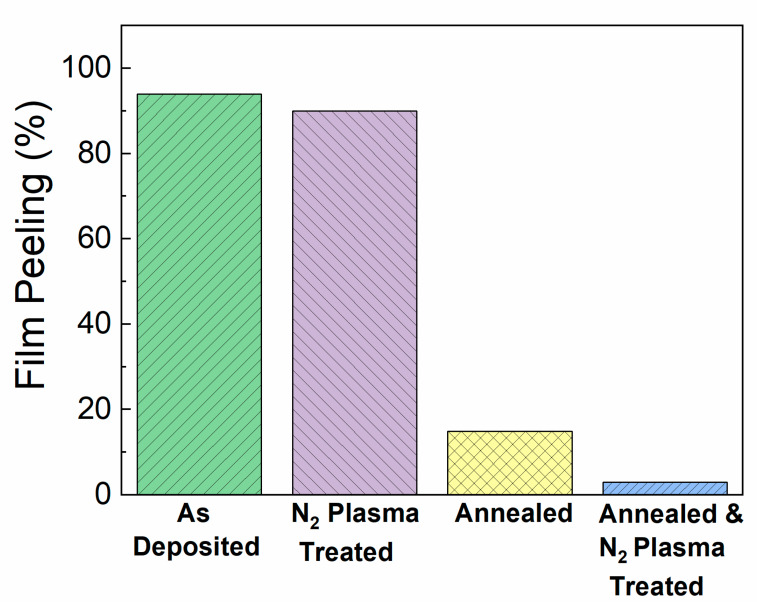
Summary of how N_2_ plasma treatment and post-deposition annealing affect delamination and bubbling of SiC-coated fluorapatite glass-ceramics after immersion in a pH 10 buffer solution at 80 °C for 30 days.

The surface area analysis after immersion in pH 10 solution was repeated for all the conditions studied and is summarized in Figure 6. The percentage of the SiC surface area that delaminated or bubbled after immersion in a pH 10 buffer solution was 94% for the as-deposited SiC, 90% plasma-treated SiC, 14.9% for the annealed SiC, and 2.9% for the plasma-treated and annealed SiC.

Based on our findings, SiC deposition parameters, as well as post-deposition processing steps, can highly influence the chemical durability of the coating. There are critical thresholds in both mass-density/porosity that dictate the diffusion of moisture and solvents through dielectric materials. King et al. observed that hermetic dielectric materials were achieved at mass densities greater than 2.0 g/cm^3^ and when the average pore diameter was less than twice the molecular diameter of water [49]. With regards to the SiC films investigated in this study, both the annealed-only SiC and the plasma-treated SiC followed by an annealing step exhibited densities greater than 2.0 g/cm^3^ and demonstrated the least film delamination. Therefore, although N_2_ plasma treatment does help reduce film delamination, the SiC density and corresponding pore size is the driving factor for the film’s diffusive properties. The lowest peeling percentage for the SiC samples that were annealed and plasma-treated are primarily due to an increased density of the SiC from annealing and secondarily due to the N_2_ plasma forming a SiO_2_ cap layer that assisted in surface pore sealing. These delamination results are valid only for the conditions studied (constant immersion, pH 10, 80 °C) and that measurements performed at different temperatures and pH levels could yield varied results. However, since we chose the conditions that are the most corrosive to SiC in this report, we believe other conditions tested would be less aggressive. Regardless, the general trends between solvent/moisture diffusion and porosity, mass density, and surface conditions are expected to be maintained regardless of the experimental condition.

## 4. Conclusions

We have demonstrated that post-deposition processing steps such as annealing and N_2_ plasma treatment can be utilized to improve the chemical durability of SiC-based protective coatings. Film stress measurements showed that post-deposition annealing at 400 °C for 12 h significantly reduced the intrinsic stress in the SiC film, whereas N_2_ plasma treatment did not affect the film stress of the SiC. The use of FTIR and XPS showed that post-deposition annealing broke hydrogen-related bonds within the film but minimally affected the SiC surface, whereas N_2_ plasma treatment did not affect the SiC bulk chemistry but sealed surface pores by forming an oxidized surface. XRR measurements confirmed that post-deposition annealing densified the SiC coating, while N_2_ plasma treatment slightly reduced the SiC density. When comparing as-deposited SiC to SiC that had been treated with an N_2_ plasma followed by an annealing step, film delamination was reduced from 94% to 2.9% after immersion in a pH 10 buffer solution at 80 °C for 30 days. Post-deposition annealing had the largest influence on chemical durability improvement as the mass-density of SiC is directly correlated to the porosity. For hermetic moisture barrier performance, SiC with a density > 2.0 g/cm^3^ is desirable. Further work should be done to study SiC film delamination effects in systems that cycle pH from acidic to basic, which is more representative of the final environment of these glass-ceramics.

## Figures and Tables

**Table 1:** Atomic concentration of SiC after each processing condition.

Element	As Deposited	Annealed	N_2_ Plasma Treated
Silicon (Si 2p)	36.01	32.29	32.03
Oxygen (O 1s)	8.97	33.70	59.11
Nitrogen (N 1s)	2.42	1.13	1.90
Carbon (C 1s)	50.68	32.88	6.97

**Table 2:** Density of SiC film determined by XRR.

SiC Treatment	Density (g/cm^3^)
As deposited	1.99
Nitrogen plasma	1.92
Annealed and Nitrogen plasma	2.06
Annealed	2.11
